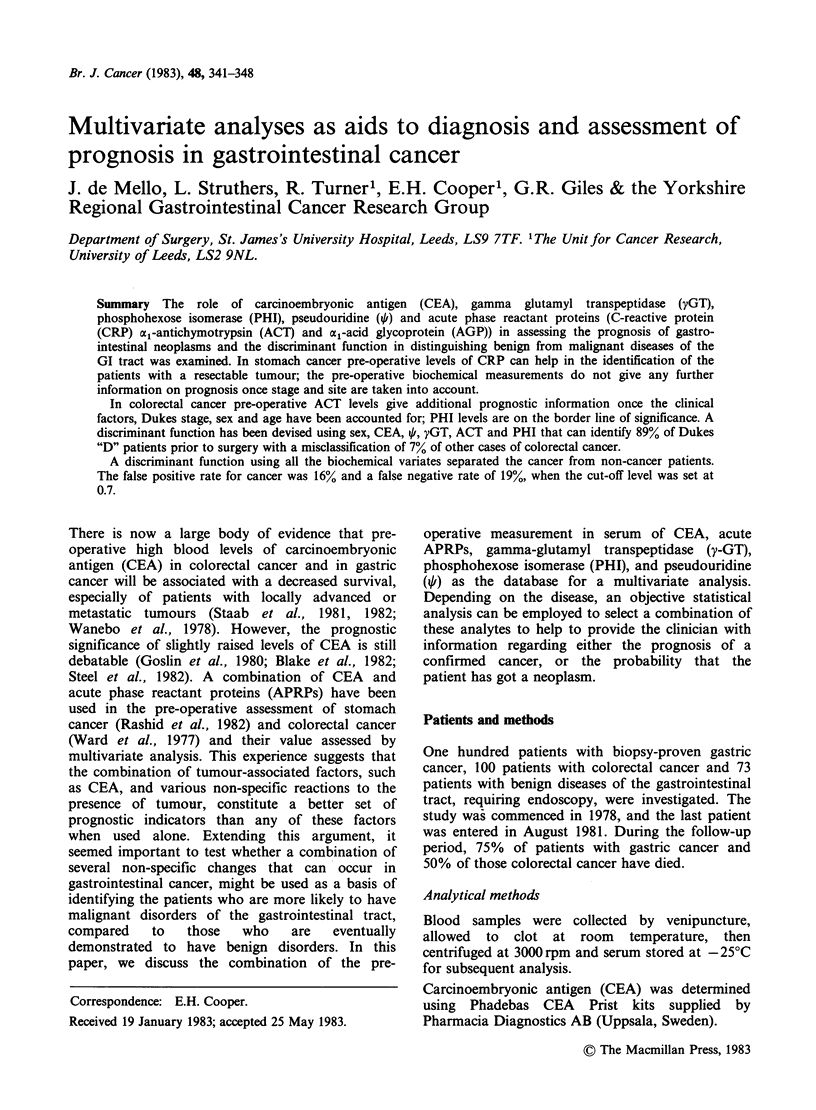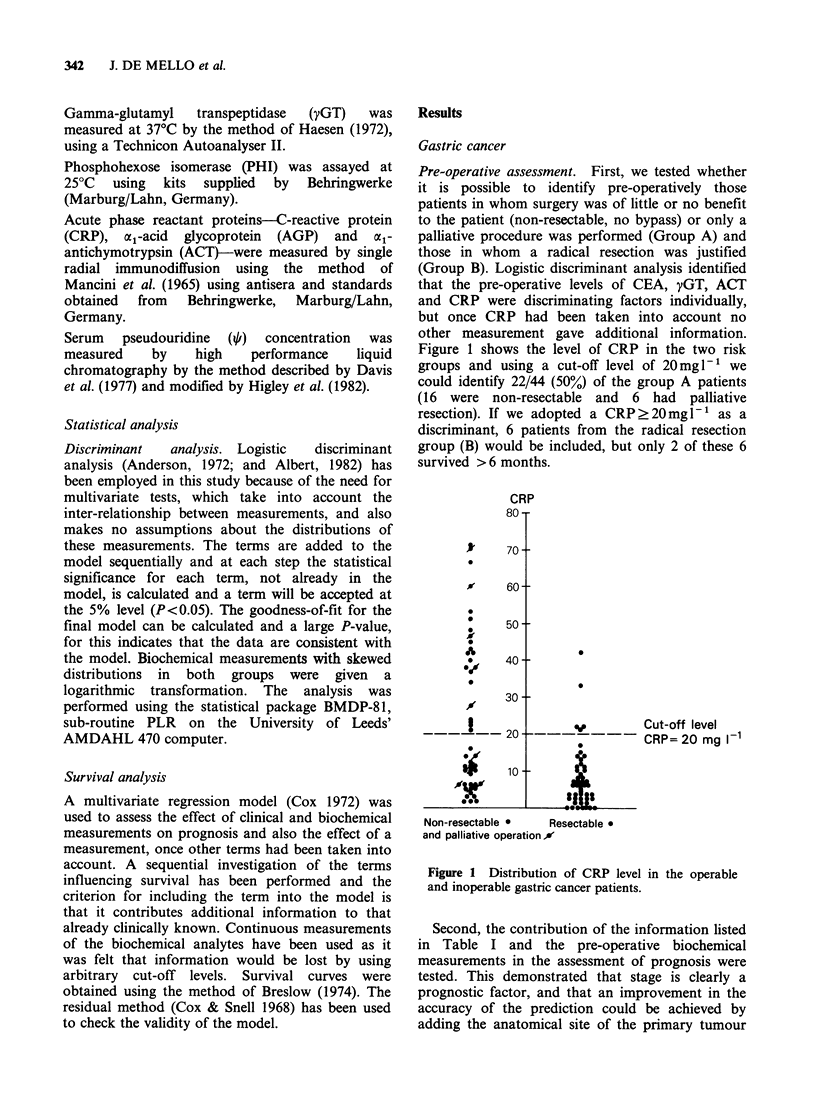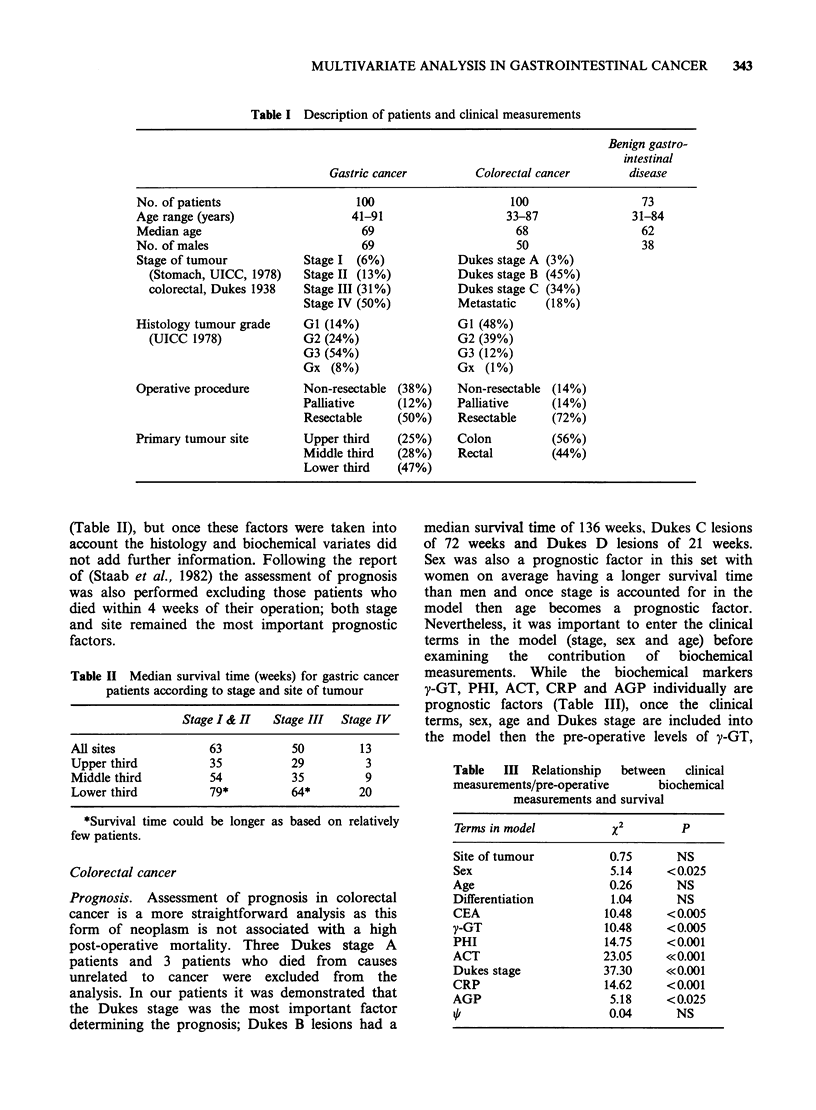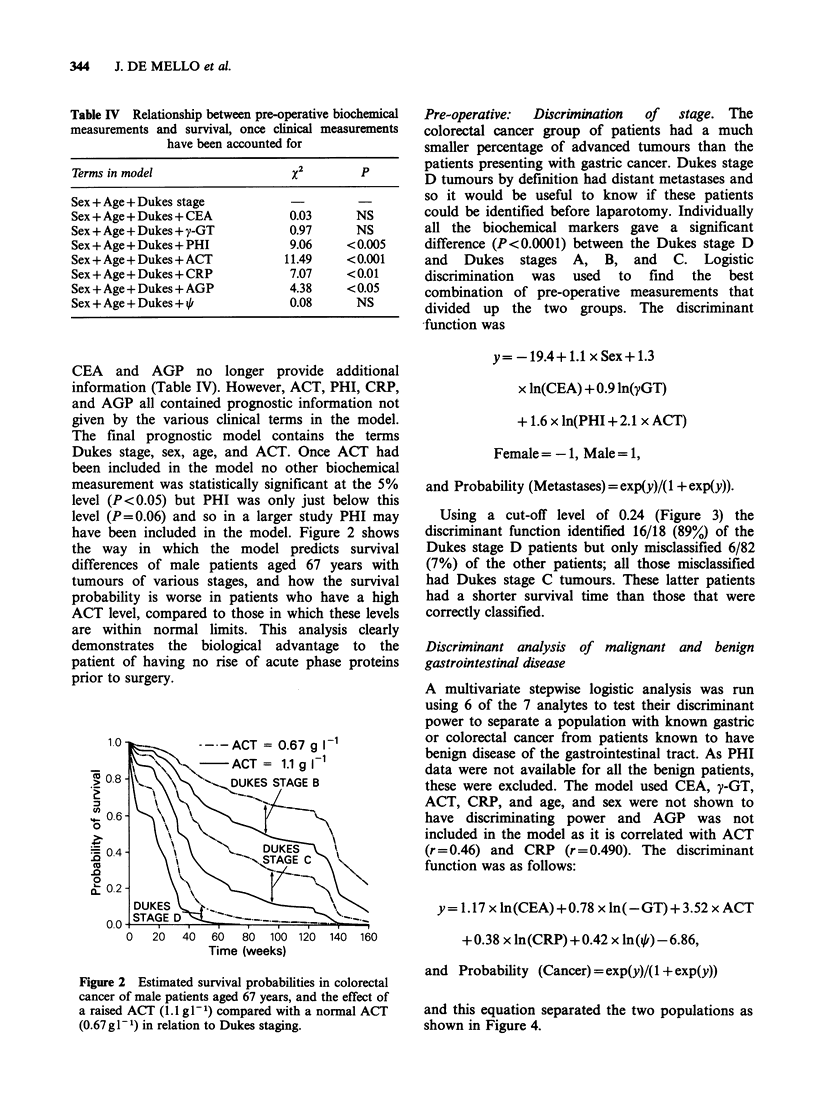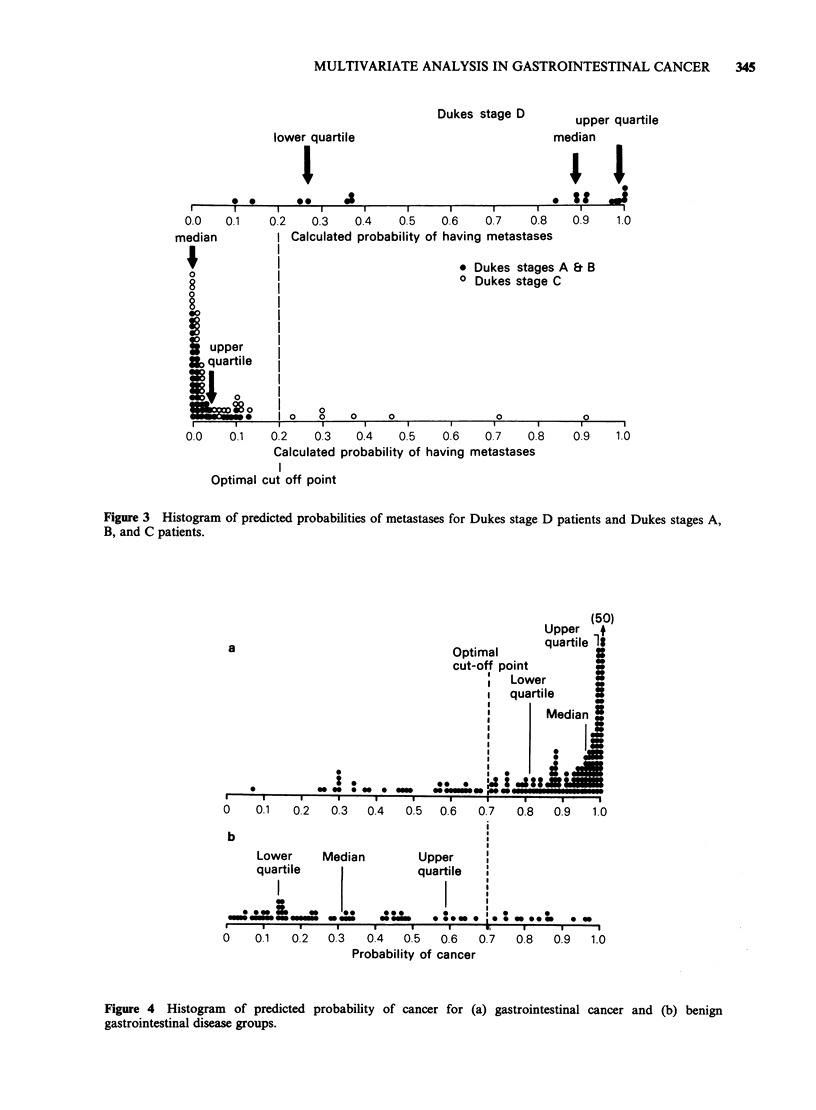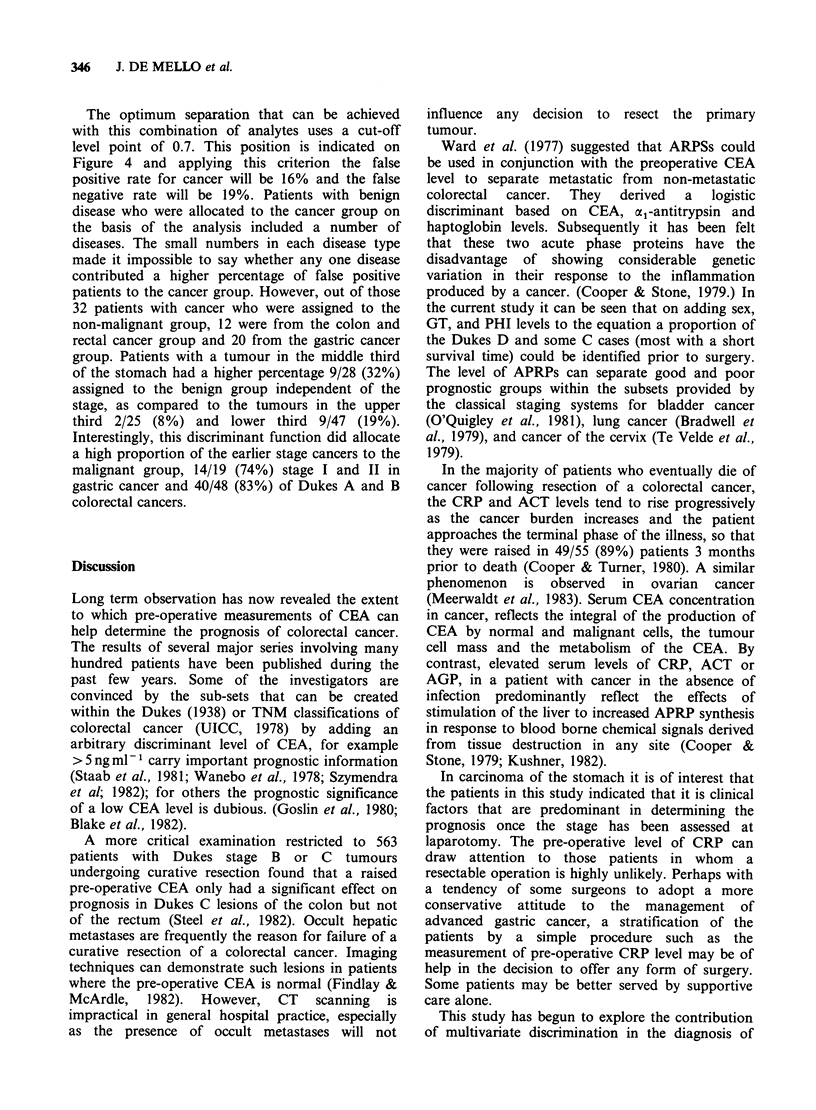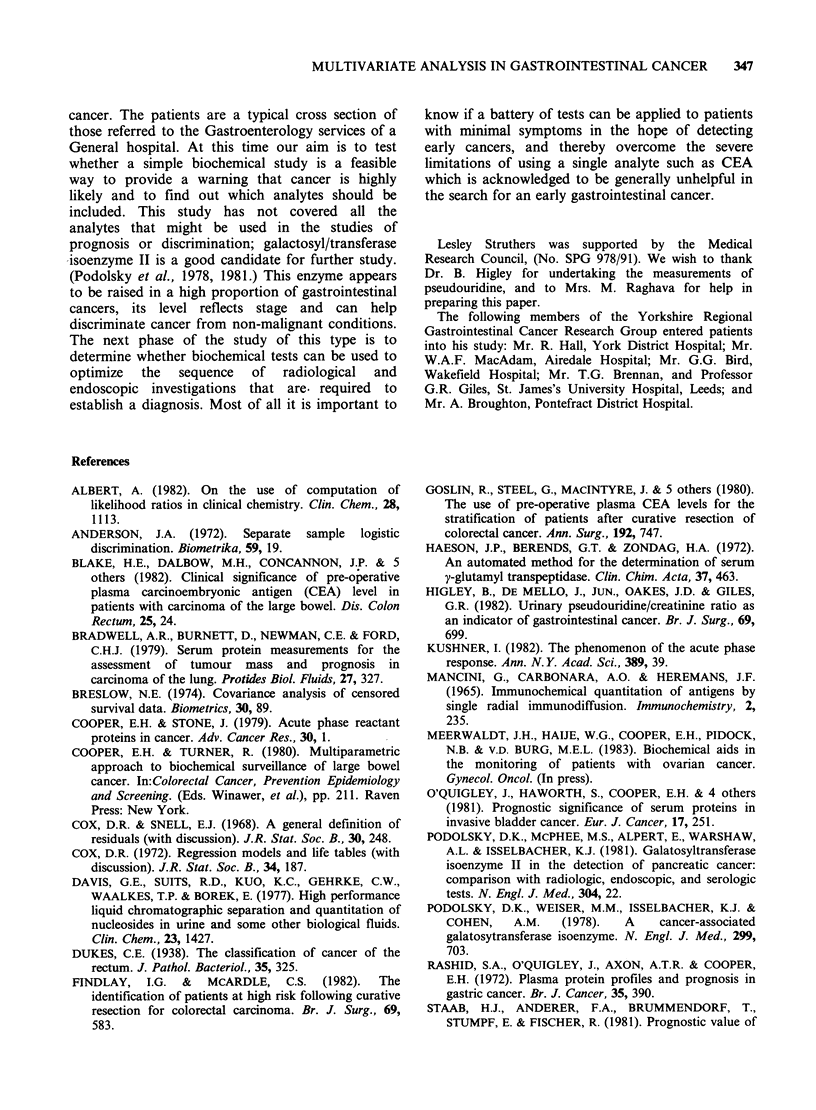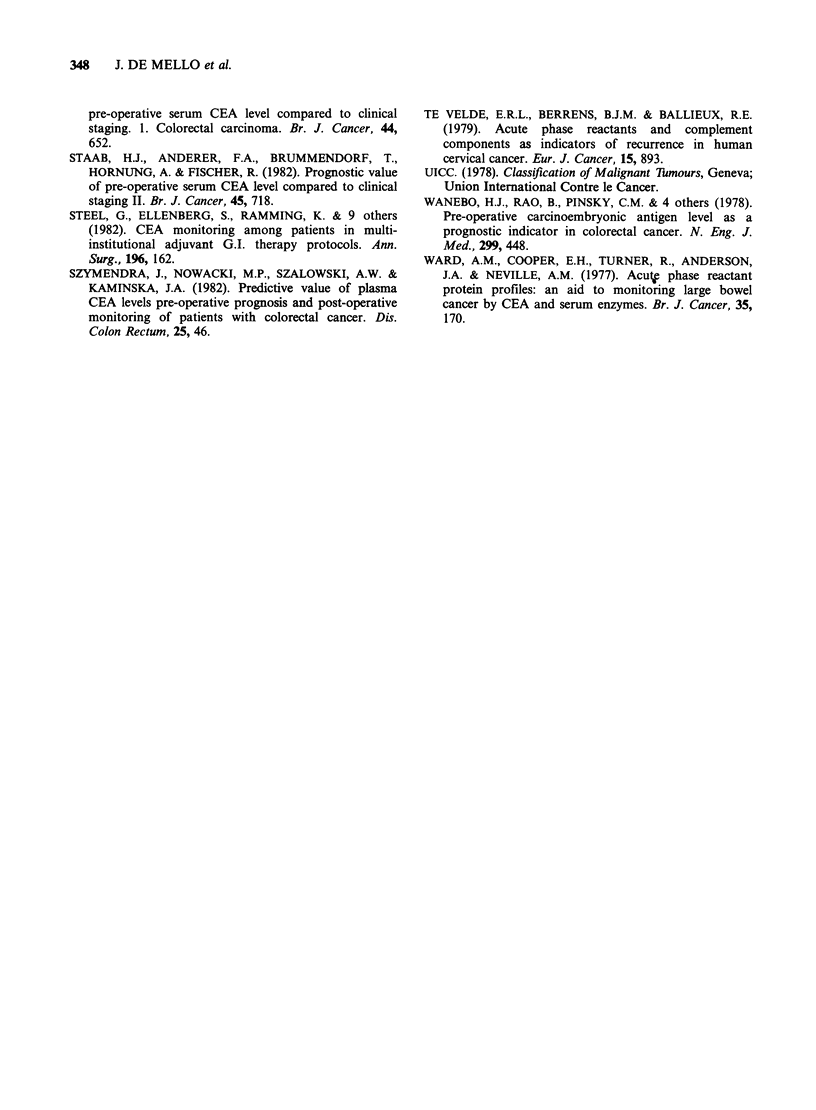# Multivariate analyses as aids to diagnosis and assessment of prognosis in gastrointestinal cancer.

**DOI:** 10.1038/bjc.1983.198

**Published:** 1983-09

**Authors:** J. de Mello, L. Struthers, R. Turner, E. H. Cooper, G. R. Giles

## Abstract

The role of carcinoembryonic antigen (CEA), gamma glutamyl transpeptidase (gamma GT), phosphohexose isomerase (PHI), pseudouridine (psi) and acute phase reactant proteins (C-reactive protein (CRP) alpha 1-antichymotrypsin (ACT) and alpha 1-acid glycoprotein (AGP] in assessing the prognosis of gastrointestinal neoplasms and the discriminant function in distinguishing benign from malignant diseases of the GI tract was examined. In stomach cancer pre-operative levels of CRP can help in the identification of the patients with a resectable tumour; the pre-operative biochemical measurements do not give any further information on prognosis once stage and site are taken into account. In colorectal cancer pre-operative ACT levels give additional prognostic information once the clinical factors, Dukes stage, sex and age have been accounted for; PHI levels are on the border line of significance. A discriminant function has been devised using sex, CEA, psi, gamma GT, ACT and PHI that can identify 89% of Dukes "D" patients prior to surgery with a misclassification of 7% of other cases of colorectal cancer. A discriminant function using all the biochemical variates separated the cancer from non-cancer patients. The false positive rate for cancer was 16% and a false negative rate of 19%, when the cut-off level was set at 0.7.


*Br. J. Cancer* (1983), **48**, 341–348

### Multivariate analyses as aids to diagnosis and assessment of prognosis in gastrointestinal cancer

J. de Mello, L. Struthers, R. Turner[1], E.H. Cooper[1], G.R. Giles & the Yorkshire Regional Gastrointestinal Cancer Research Group

*Department of Surgery, St. James's University Hospital, Leeds, LS9 7TF. [1]The Unit for Cancer Research, University of Leeds, LS2 9NL.*

**Summary** The role of carcinoembryonic antigen (CEA), gamma glutamyl transpeptidase ($\gamma$GT), phosphohexose isomerase (PHI), pseudouridine ($\psi$) and acute phase reactant proteins (C-reactive protein (CRP) $\alpha_1$-antichymotrypsin (ACT) and $\alpha_1$-acid glycoprotein (AGP)) in assessing the prognosis of gastrointestinal neoplasms and the discriminant function in distinguishing benign from malignant diseases of the GI tract was examined. In stomach cancer pre-operative levels of CRP can help in the identification of the patients with a resectable tumour; the pre-operative biochemical measurements do not give any further information on prognosis once stage and site are taken into account.

In colorectal cancer pre-operative ACT levels give additional prognostic information once the clinical factors, Dukes stage, sex and age have been accounted for; PHI levels are on the border line of significance. A discriminant function has been devised using sex, CEA, $\psi$, $\gamma$GT, ACT and PHI that can identify 89% of Dukes "D" patients prior to surgery with a misclassification of 7% of other cases of colorectal cancer.

A discriminant function using all the biochemical variates separated the cancer from non-cancer patients. The false positive rate for cancer was 16% and a false negative rate of 19%, when the cut-off level was set at 0.7.

There is now a large body of evidence that pre-operative high blood levels of carcinoembryonic antigen (CEA) in colorectal cancer and in gastric cancer will be associated with a decreased survival, especially of patients with locally advanced or metastatic tumours (Staab *et al.*, 1981, 1982; Wanebo *et al.*, 1978). However, the prognostic significance of slightly raised levels of CEA is still debatable (Goslin *et al.*, 1980; Blake *et al.*, 1982; Steel *et al.*, 1982). A combination of CEA and acute phase reactant proteins (APRPs) have been used in the pre-operative assessment of stomach cancer (Rashid *et al.*, 1982) and colorectal cancer (Ward *et al.*, 1977) and their value assessed by multivariate analysis. This experience suggests that the combination of tumour-associated factors, such as CEA, and various non-specific reactions to the presence of tumour, constitute a better set of prognostic indicators than any of these factors when used alone. Extending this argument, it seemed important to test whether a combination of several non-specific changes that can occur in gastrointestinal cancer, might be used as a basis of identifying the patients who are more likely to have malignant disorders of the gastrointestinal tract, compared to those who are eventually demonstrated to have benign disorders. In this paper, we discuss the combination of the pre-operative measurement in serum of CEA, acute APRPs, gamma-glutamyl transpeptidase ($\gamma$-GT), phosphohexose isomerase (PHI), and pseudouridine ($\psi$) as the database for a multivariate analysis. Depending on the disease, an objective statistical analysis can be employed to select a combination of these analytes to help to provide the clinician with information regarding either the prognosis of a confirmed cancer, or the probability that the patient has got a neoplasm.

Correspondence: E.H. Cooper.

Received 19 January 1983; accepted 25 May 1983.

### Patients and methods

One hundred patients with biopsy-proven gastric cancer, 100 patients with colorectal cancer and 73 patients with benign diseases of the gastrointestinal tract, requiring endoscopy, were investigated. The study was commenced in 1978, and the last patient was entered in August 1981. During the follow-up period, 75% of patients with gastric cancer and 50% of those colorectal cancer have died.

### *Analytical methods*

Blood samples were collected by venipuncture, allowed to clot at room temperature, then centrifuged at 3000 rpm and serum stored at −25°C for subsequent analysis.

Carcinoembryonic antigen (CEA) was determined using Phadebas CEA Prist kits supplied by Pharmacia Diagnostics AB (Uppsala, Sweden).

© The Macmillan Press, 1983

Gamma-glutamyl transpeptidase ($\gamma$GT) was measured at 37°C by the method of Haesen (1972), using a Technicon Autoanalyser II.

Phosphohexose isomerase (PHI) was assayed at 25°C using kits supplied by Behringwerke (Marburg/Lahn, Germany).

Acute phase reactant proteins—C-reactive protein (CRP), $\alpha_1$-acid glycoprotein (AGP) and $\alpha_1$-antichymotrypsin (ACT)—were measured by single radial immunodiffusion using the method of Mancini et al. (1965) using antisera and standards obtained from Behringwerke, Marburg/Lahn, Germany.

Serum pseudouridine ($\psi$) concentration was measured by high performance liquid chromatography by the method described by Davis et al. (1977) and modified by Higley et al. (1982).

### Statistical analysis

*Discriminant analysis.* Logistic discriminant analysis (Anderson, 1972; and Albert, 1982) has been employed in this study because of the need for multivariate tests, which take into account the inter-relationship between measurements, and also makes no assumptions about the distributions of these measurements. The terms are added to the model sequentially and at each step the statistical significance for each term, not already in the model, is calculated and a term will be accepted at the 5% level ($P<0.05$). The goodness-of-fit for the final model can be calculated and a large $P$-value, for this indicates that the data are consistent with the model. Biochemical measurements with skewed distributions in both groups were given a logarithmic transformation. The analysis was performed using the statistical package BMDP-81, sub-routine PLR on the University of Leeds' AMDAHL 470 computer.

### Survival analysis

A multivariate regression model (Cox 1972) was used to assess the effect of clinical and biochemical measurements on prognosis and also the effect of a measurement, once other terms had been taken into account. A sequential investigation of the terms influencing survival has been performed and the criterion for including the term into the model is that it contributes additional information to that already clinically known. Continuous measurements of the biochemical analytes have been used as it was felt that information would be lost by using arbitrary cut-off levels. Survival curves were obtained using the method of Breslow (1974). The residual method (Cox & Snell 1968) has been used to check the validity of the model.

### Results

### Gastric cancer

*Pre-operative assessment.* First, we tested whether it is possible to identify pre-operatively those patients in whom surgery was of little or no benefit to the patient (non-resectable, no bypass) or only a palliative procedure was performed (Group A) and those in whom a radical resection was justified (Group B). Logistic discriminant analysis identified that the pre-operative levels of CEA, $\gamma$GT, ACT and CRP were discriminating factors individually, but once CRP had been taken into account no other measurement gave additional information. Figure 1 shows the level of CRP in the two risk groups and using a cut-off level of $20\,mg\,l^{-1}$ we could identify 22/44 (50%) of the group A patients (16 were non-resectable and 6 had palliative resection). If we adopted a $CRP \geq 20\,mg\,l^{-1}$ as a discriminant, 6 patients from the radical resection group (B) would be included, but only 2 of these 6 survived >6 months.

**Figure 1** Distribution of CRP level in the operable and inoperable gastric cancer patients.

Second, the contribution of the information listed in Table I and the pre-operative biochemical measurements in the assessment of prognosis were tested. This demonstrated that stage is clearly a prognostic factor, and that an improvement in the accuracy of the prediction could be achieved by adding the anatomical site of the primary tumour

**Table I** Description of patients and clinical measurements

| | *Gastric cancer* | *Colorectal cancer* | *Benign gastro-intestinal disease* |
|---|---|---|---|
| No. of patients | 100 | 100 | 73 |
| Age range (years) | 41–91 | 33–87 | 31–84 |
| Median age | 69 | 68 | 62 |
| No. of males | 69 | 50 | 38 |
| Stage of tumour (Stomach, UICC, 1978) colorectal, Dukes 1938 | Stage I (6%)<br>Stage II (13%)<br>Stage III (31%)<br>Stage IV (50%) | Dukes stage A (3%)<br>Dukes stage B (45%)<br>Dukes stage C (34%)<br>Metastatic (18%) | |
| Histology tumour grade (UICC 1978) | G1 (14%)<br>G2 (24%)<br>G3 (54%)<br>Gx (8%) | G1 (48%)<br>G2 (39%)<br>G3 (12%)<br>Gx (1%) | |
| Operative procedure | Non-resectable (38%)<br>Palliative (12%)<br>Resectable (50%) | Non-resectable (14%)<br>Palliative (14%)<br>Resectable (72%) | |
| Primary tumour site | Upper third (25%)<br>Middle third (28%)<br>Lower third (47%) | Colon (56%)<br>Rectal (44%) | |

(Table II), but once these factors were taken into account the histology and biochemical variates did not add further information. Following the report of (Staab *et al.*, 1982) the assessment of prognosis was also performed excluding those patients who died within 4 weeks of their operation; both stage and site remained the most important prognostic factors.

**Table II** Median survival time (weeks) for gastric cancer patients according to stage and site of tumour

| | *Stage I & II* | *Stage III* | *Stage IV* |
|---|---|---|---|
| All sites | 63 | 50 | 13 |
| Upper third | 35 | 29 | 3 |
| Middle third | 54 | 35 | 9 |
| Lower third | 79* | 64* | 20 |

*Survival time could be longer as based on relatively few patients.

### *Colorectal cancer*

*Prognosis.* Assessment of prognosis in colorectal cancer is a more straightforward analysis as this form of neoplasm is not associated with a high post-operative mortality. Three Dukes stage A patients and 3 patients who died from causes unrelated to cancer were excluded from the analysis. In our patients it was demonstrated that the Dukes stage was the most important factor determining the prognosis; Dukes B lesions had a median survival time of 136 weeks, Dukes C lesions of 72 weeks and Dukes D lesions of 21 weeks. Sex was also a prognostic factor in this set with women on average having a longer survival time than men and once stage is accounted for in the model then age becomes a prognostic factor. Nevertheless, it was important to enter the clinical terms in the model (stage, sex and age) before examining the contribution of biochemical measurements. While the biochemical markers $\gamma$-GT, PHI, ACT, CRP and AGP individually are prognostic factors (Table III), once the clinical terms, sex, age and Dukes stage are included into the model then the pre-operative levels of $\gamma$-GT,

**Table III** Relationship between clinical measurements/pre-operative biochemical measurements and survival

| *Terms in model* | $\chi^2$ | *P* |
|---|---|---|
| Site of tumour | 0.75 | NS |
| Sex | 5.14 | <0.025 |
| Age | 0.26 | NS |
| Differentiation | 1.04 | NS |
| CEA | 10.48 | <0.005 |
| $\gamma$-GT | 10.48 | <0.005 |
| PHI | 14.75 | <0.001 |
| ACT | 23.05 | ≪0.001 |
| Dukes stage | 37.30 | ≪0.001 |
| CRP | 14.62 | <0.001 |
| AGP | 5.18 | <0.025 |
| $\psi$ | 0.04 | NS |

**Table IV** Relationship between pre-operative biochemical measurements and survival, once clinical measurements have been accounted for

| *Terms in model* | $\chi^2$ | *P* |
|---|---|---|
| Sex + Age + Dukes stage | — | — |
| Sex + Age + Dukes + CEA | 0.03 | NS |
| Sex + Age + Dukes + $\gamma$-GT | 0.97 | NS |
| Sex + Age + Dukes + PHI | 9.06 | <0.005 |
| Sex + Age + Dukes + ACT | 11.49 | <0.001 |
| Sex + Age + Dukes + CRP | 7.07 | <0.01 |
| Sex + Age + Dukes + AGP | 4.38 | <0.05 |
| Sex + Age + Dukes + $\psi$ | 0.08 | NS |

CEA and AGP no longer provide additional information (Table IV). However, ACT, PHI, CRP, and AGP all contained prognostic information not given by the various clinical terms in the model. The final prognostic model contains the terms Dukes stage, sex, age, and ACT. Once ACT had been included in the model no other biochemical measurement was statistically significant at the 5% level ($P<0.05$) but PHI was only just below this level ($P=0.06$) and so in a larger study PHI may have been included in the model. Figure 2 shows the way in which the model predicts survival differences of male patients aged 67 years with tumours of various stages, and how the survival probability is worse in patients who have a high ACT level, compared to those in which these levels are within normal limits. This analysis clearly demonstrates the biological advantage to the patient of having no rise of acute phase proteins prior to surgery.

**Figure 2** Estimated survival probabilities in colorectal cancer of male patients aged 67 years, and the effect of a raised ACT ($1.1\,g\,l^{-1}$) compared with a normal ACT ($0.67\,g\,l^{-1}$) in relation to Dukes staging.

*Pre-operative: Discrimination of stage.* The colorectal cancer group of patients had a much smaller percentage of advanced tumours than the patients presenting with gastric cancer. Dukes stage D tumours by definition had distant metastases and so it would be useful to know if these patients could be identified before laparotomy. Individually all the biochemical markers gave a significant difference ($P<0.0001$) between the Dukes stage D and Dukes stages A, B, and C. Logistic discrimination was used to find the best combination of pre-operative measurements that divided up the two groups. The discriminant function was

$$y = -19.4 + 1.1 \times \text{Sex} + 1.3 \times \ln(\text{CEA}) + 0.9\ln(\gamma\text{GT}) + 1.6 \times \ln(\text{PHI} + 2.1 \times \text{ACT})$$

$$\text{Female} = -1,\ \text{Male} = 1,$$

and Probability (Metastases) $= \exp(y)/(1+\exp(y))$.

Using a cut-off level of 0.24 (Figure 3) the discriminant function identified 16/18 (89%) of the Dukes stage D patients but only misclassified 6/82 (7%) of the other patients; all those misclassified had Dukes stage C tumours. These latter patients had a shorter survival time than those that were correctly classified.

*Discriminant analysis of malignant and benign gastrointestinal disease*

A multivariate stepwise logistic analysis was run using 6 of the 7 analytes to test their discriminant power to separate a population with known gastric or colorectal cancer from patients known to have benign disease of the gastrointestinal tract. As PHI data were not available for all the benign patients, these were excluded. The model used CEA, $\gamma$-GT, ACT, CRP, and age, and sex were not shown to have discriminating power and AGP was not included in the model as it is correlated with ACT ($r=0.46$) and CRP ($r=0.490$). The discriminant function was as follows:

$$y = 1.17 \times \ln(\text{CEA}) + 0.78 \times \ln(-\text{GT}) + 3.52 \times \text{ACT} + 0.38 \times \ln(\text{CRP}) + 0.42 \times \ln(\psi) - 6.86,$$

and Probability (Cancer) $= \exp(y)/(1+\exp(y))$

and this equation separated the two populations as shown in Figure 4.

**Figure 3** Histogram of predicted probabilities of metastases for Dukes stage D patients and Dukes stages A, B, and C patients.

**Figure 4** Histogram of predicted probability of cancer for (a) gastrointestinal cancer and (b) benign gastrointestinal disease groups.

The optimum separation that can be achieved with this combination of analytes uses a cut-off level point of 0.7. This position is indicated on Figure 4 and applying this criterion the false positive rate for cancer will be 16% and the false negative rate will be 19%. Patients with benign disease who were allocated to the cancer group on the basis of the analysis included a number of diseases. The small numbers in each disease type made it impossible to say whether any one disease contributed a higher percentage of false positive patients to the cancer group. However, out of those 32 patients with cancer who were assigned to the non-malignant group, 12 were from the colon and rectal cancer group and 20 from the gastric cancer group. Patients with a tumour in the middle third of the stomach had a higher percentage 9/28 (32%) assigned to the benign group independent of the stage, as compared to the tumours in the upper third 2/25 (8%) and lower third 9/47 (19%). Interestingly, this discriminant function did allocate a high proportion of the earlier stage cancers to the malignant group, 14/19 (74%) stage I and II in gastric cancer and 40/48 (83%) of Dukes A and B colorectal cancers.

### Discussion

Long term observation has now revealed the extent to which pre-operative measurements of CEA can help determine the prognosis of colorectal cancer. The results of several major series involving many hundred patients have been published during the past few years. Some of the investigators are convinced by the sub-sets that can be created within the Dukes (1938) or TNM classifications of colorectal cancer (UICC, 1978) by adding an arbitrary discriminant level of CEA, for example $>5\,ng\,ml^{-1}$ carry important prognostic information (Staab *et al.*, 1981; Wanebo *et al.*, 1978; Szymendra *et al*; 1982); for others the prognostic significance of a low CEA level is dubious. (Goslin *et al.*, 1980; Blake *et al.*, 1982).

A more critical examination restricted to 563 patients with Dukes stage B or C tumours undergoing curative resection found that a raised pre-operative CEA only had a significant effect on prognosis in Dukes C lesions of the colon but not of the rectum (Steel *et al.*, 1982). Occult hepatic metastases are frequently the reason for failure of a curative resection of a colorectal cancer. Imaging techniques can demonstrate such lesions in patients where the pre-operative CEA is normal (Findlay & McArdle, 1982). However, CT scanning is impractical in general hospital practice, especially as the presence of occult metastases will not influence any decision to resect the primary tumour.

Ward *et al.* (1977) suggested that ARPSs could be used in conjunction with the preoperative CEA level to separate metastatic from non-metastatic colorectal cancer. They derived a logistic discriminant based on CEA, $\alpha_1$-antitrypsin and haptoglobin levels. Subsequently it has been felt that these two acute phase proteins have the disadvantage of showing considerable genetic variation in their response to the inflammation produced by a cancer. (Cooper & Stone, 1979.) In the current study it can be seen that on adding sex, GT, and PHI levels to the equation a proportion of the Dukes D and some C cases (most with a short survival time) could be identified prior to surgery. The level of APRPs can separate good and poor prognostic groups within the subsets provided by the classical staging systems for bladder cancer (O'Quigley *et al.*, 1981), lung cancer (Bradwell *et al.*, 1979), and cancer of the cervix (Te Velde *et al.*, 1979).

In the majority of patients who eventually die of cancer following resection of a colorectal cancer, the CRP and ACT levels tend to rise progressively as the cancer burden increases and the patient approaches the terminal phase of the illness, so that they were raised in 49/55 (89%) patients 3 months prior to death (Cooper & Turner, 1980). A similar phenomenon is observed in ovarian cancer (Meerwaldt *et al.*, 1983). Serum CEA concentration in cancer, reflects the integral of the production of CEA by normal and malignant cells, the tumour cell mass and the metabolism of the CEA. By contrast, elevated serum levels of CRP, ACT or AGP, in a patient with cancer in the absence of infection predominantly reflect the effects of stimulation of the liver to increased APRP synthesis in response to blood borne chemical signals derived from tissue destruction in any site (Cooper & Stone, 1979; Kushner, 1982).

In carcinoma of the stomach it is of interest that the patients in this study indicated that it is clinical factors that are predominant in determining the prognosis once the stage has been assessed at laparotomy. The pre-operative level of CRP can draw attention to those patients in whom a resectable operation is highly unlikely. Perhaps with a tendency of some surgeons to adopt a more conservative attitude to the management of advanced gastric cancer, a stratification of the patients by a simple procedure such as the measurement of pre-operative CRP level may be of help in the decision to offer any form of surgery. Some patients may be better served by supportive care alone.

This study has begun to explore the contribution of multivariate discrimination in the diagnosis of cancer. The patients are a typical cross section of those referred to the Gastroenterology services of a General hospital. At this time our aim is to test whether a simple biochemical study is a feasible way to provide a warning that cancer is highly likely and to find out which analytes should be included. This study has not covered all the analytes that might be used in the studies of prognosis or discrimination; galactosyl/transferase isoenzyme II is a good candidate for further study. (Podolsky *et al.*, 1978, 1981.) This enzyme appears to be raised in a high proportion of gastrointestinal cancers, its level reflects stage and can help discriminate cancer from non-malignant conditions. The next phase of the study of this type is to determine whether biochemical tests can be used to optimize the sequence of radiological and endoscopic investigations that are required to establish a diagnosis. Most of all it is important to know if a battery of tests can be applied to patients with minimal symptoms in the hope of detecting early cancers, and thereby overcome the severe limitations of using a single analyte such as CEA which is acknowledged to be generally unhelpful in the search for an early gastrointestinal cancer.

Lesley Struthers was supported by the Medical Research Council, (No. SPG 978/91). We wish to thank Dr. B. Higley for undertaking the measurements of pseudouridine, and to Mrs. M. Raghava for help in preparing this paper.

The following members of the Yorkshire Regional Gastrointestinal Cancer Research Group entered patients into his study: Mr. R. Hall, York District Hospital; Mr. W.A.F. MacAdam, Airedale Hospital; Mr. G.G. Bird, Wakefield Hospital; Mr. T.G. Brennan, and Professor G.R. Giles, St. James's University Hospital, Leeds; and Mr. A. Broughton, Pontefract District Hospital.

### References

ALBERT, A. (1982). On the use of computation of likelihood ratios in clinical chemistry. *Clin. Chem.*, **28**, 1113.

ANDERSON, J.A. (1972). Separate sample logistic discrimination. *Biometrika*, **59**, 19.

BLAKE, H.E., DALBOW, M.H., CONCANNON, J.P. & 5 others (1982). Clinical significance of pre-operative plasma carcinoembryonic antigen (CEA) level in patients with carcinoma of the large bowel. *Dis. Colon Rectum*, **25**, 24.

BRADWELL, A.R., BURNETT, D., NEWMAN, C.E. & FORD, C.H.J. (1979). Serum protein measurements for the assessment of tumour mass and prognosis in carcinoma of the lung. *Protides Biol. Fluids*, **27**, 327.

BRESLOW, N.E. (1974). Covariance analysis of censored survival data. *Biometrics*, **30**, 89.

COOPER, E.H. & STONE, J. (1979). Acute phase reactant proteins in cancer. *Adv. Cancer Res.*, **30**, 1.

COOPER, E.H. & TURNER, R. (1980). Multiparametric approach to biochemical surveillance of large bowel cancer. In:*Colorectal Cancer, Prevention Epidemiology and Screening*. (Eds. Winawer, *et al.*), pp. 211. Raven Press: New York.

COX, D.R. & SNELL, E.J. (1968). A general definition of residuals (with discussion). *J.R. Stat. Soc. B.*, **30**, 248.

COX, D.R. (1972). Regression models and life tables (with discussion). *J.R. Stat. Soc. B.*, **34**, 187.

DAVIS, G.E., SUITS, R.D., KUO, K.C., GEHRKE, C.W., WAALKES, T.P. & BOREK, E. (1977). High performance liquid chromatographic separation and quantitation of nucleosides in urine and some other biological fluids. *Clin. Chem.*, **23**, 1427.

DUKES, C.E. (1938). The classification of cancer of the rectum. *J. Pathol. Bacteriol.*, **35**, 325.

FINDLAY, I.G. & MCARDLE, C.S. (1982). The identification of patients at high risk following curative resection for colorectal carcinoma. *Br. J. Surg.*, **69**, 583.

GOSLIN, R., STEEL, G., MACINTYRE, J. & 5 others (1980). The use of pre-operative plasma CEA levels for the stratification of patients after curative resection of colorectal cancer. *Ann. Surg.*, **192**, 747.

HAESON, J.P., BERENDS, G.T. & ZONDAG, H.A. (1972). An automated method for the determination of serum γ-glutamyl transpeptidase. *Clin. Chim. Acta*, **37**, 463.

HIGLEY, B., DE MELLO, J., JUN., OAKES, J.D. & GILES, G.R. (1982). Urinary pseudouridine/creatinine ratio as an indicator of gastrointestinal cancer. *Br. J. Surg.*, **69**, 699.

KUSHNER, I. (1982). The phenomenon of the acute phase response. *Ann. N.Y. Acad. Sci.*, **389**, 39.

MANCINI, G., CARBONARA, A.O. & HEREMANS, J.F. (1965). Immunochemical quantitation of antigens by single radial immunodiffusion. *Immunochemistry*, **2**, 235.

MEERWALDT, J.H., HAIJE, W.G., COOPER, E.H., PIDOCK, N.B. & V.D. BURG, M.E.L. (1983). Biochemical aids in the monitoring of patients with ovarian cancer. *Gynecol. Oncol.* (In press).

O'QUIGLEY, J., HAWORTH, S., COOPER, E.H. & 4 others (1981). Prognostic significance of serum proteins in invasive bladder cancer. *Eur. J. Cancer*, **17**, 251.

PODOLSKY, D.K., MCPHEE, M.S., ALPERT, E., WARSHAW, A.L. & ISSELBACHER, K.J. (1981). Galatosyltransferase isoenzyme II in the detection of pancreatic cancer: comparison with radiologic, endoscopic, and serologic tests. *N. Engl. J. Med.*, **304**, 22.

PODOLSKY, D.K., WEISER, M.M., ISSELBACHER, K.J. & COHEN, A.M. (1978). A cancer-associated galatosytransferase isoenzyme. *N. Engl. J. Med.*, **299**, 703.

RASHID, S.A., O'QUIGLEY, J., AXON, A.T.R. & COOPER, E.H. (1972). Plasma protein profiles and prognosis in gastric cancer. *Br. J. Cancer*, **35**, 390.

STAAB, H.J., ANDERER, F.A., BRUMMENDORF, T., STUMPF, E. & FISCHER, R. (1981). Prognostic value of pre-operative serum CEA level compared to clinical staging. 1. Colorectal carcinoma. *Br. J. Cancer*, **44**, 652.

STAAB, H.J., ANDERER, F.A., BRUMMENDORF, T., HORNUNG, A. & FISCHER, R. (1982). Prognostic value of pre-operative serum CEA level compared to clinical staging II. *Br. J. Cancer*, **45**, 718.

STEEL, G., ELLENBERG, S., RAMMING, K. & 9 others (1982). CEA monitoring among patients in multi-institutional adjuvant G.I. therapy protocols. *Ann. Surg.*, **196**, 162.

SZYMENDRA, J., NOWACKI, M.P., SZALOWSKI, A.W. & KAMINSKA, J.A. (1982). Predictive value of plasma CEA levels pre-operative prognosis and post-operative monitoring of patients with colorectal cancer. *Dis. Colon Rectum*, **25**, 46.

TE VELDE, E.R.L., BERRENS, B.J.M. & BALLIEUX, R.E. (1979). Acute phase reactants and complement components as indicators of recurrence in human cervical cancer. *Eur. J. Cancer*, **15**, 893.

UICC. (1978). *Classification of Malignant Tumours*, Geneva; Union International Contre le Cancer.

WANEBO, H.J., RAO, B., PINSKY, C.M. & 4 others (1978). Pre-operative carcinoembryonic antigen level as a prognostic indicator in colorectal cancer. *N. Eng. J. Med.*, **299**, 448.

WARD, A.M., COOPER, E.H., TURNER, R., ANDERSON, J.A. & NEVILLE, A.M. (1977). Acute phase reactant protein profiles: an aid to monitoring large bowel cancer by CEA and serum enzymes. *Br. J. Cancer*, **35**, 170.